# Determinants of induced abortion among women received maternal health care services in public hospitals of Arba Minch and Wolayita Sodo town, southern Ethiopia: unmatched case–control study

**DOI:** 10.1186/s12905-022-01695-0

**Published:** 2022-04-09

**Authors:** Mesfin Abebe, Abera Mersha, Nega Degefa, Feleke Gebremeskel, Etenesh Kefelew, Wondwosen Molla

**Affiliations:** 1 Department of Midwifery, College of Medicine and Health Sciences, Dilla University, Dilla, Ethiopia; 2 School of Nursing, College of Medicine and Health Sciences, Arba Minch University, Arba Minch, Ethiopia; 3 School of Public Health, College of Medicine and Health Sciences, Arba Minch University, Arba Minch, Ethiopia

**Keywords:** Induced abortion, Determinant, Maternal health care service, Ethiopia

## Abstract

**Background:**

About 210 million women become pregnant per year, with one out of every ten pregnancies terminating unsafely worldwide. In developing countries, unsafe induced abortion is a leading cause of maternal mortality and morbidity. In addition, the burden of public health is also greatest in developing regions. In Ethiopia, abortion was responsible for 8.6% of maternal deaths. Despite the problem's significance, little is known about the factors that lead to women terminating their pregnancies. Therefore, this study aims to identify the factors associated with having induced abortion in public hospitals of Arba Minch and Wolayita Sodo town, Southern Ethiopia.

**Methods:**

An institutional-based unmatched case–control study was conducted among 413 women from 15th April to 15th June 2021 in selected public hospitals of Arba Minch and Wolayita Sodo town, Southern Ethiopia. Cases were women who received induced abortion care services or who received post-abortion care services after being presented to the selected public hospital with an attempt of induced abortion whereas controls were women who came for maternal health care (antenatal or postnatal care) services in selected public hospitals and never had history of induced abortion. The data were collected by pretested and structured questionnaires with face-to-face interviews via Kobo Collect v3.1 mobile tools and analyzed by STATA version14. Logistic regression model was used to identify factors associated with induced abortion. In this study *P*-value less than 0.05 with 95% CI was declared a result as statistically significant.

**Results:**

In this study, 103 cases and 309 controls were participated. Urban residence (AOR = 2.33, 95%CI:1.26, 4.32), encountered first sex at age of 20–24 years (AOR = 0.51, 95%CI:0.27,0.97), multiple sexual partner (AOR = 5.47, 95%CI: 2.98,10.03), women who had one child (AOR = 0.32, 95%CI: 0.10, 0.99), and good knowledge of contraceptives (AOR = 0.12, 95%CI: 0.03, 0.46) were identified as determinants of induced abortion.

**Conclusions:**

Interventions focusing on those identified factors could probably reduce the burden and consequences of induced abortion. Sexual and reproductive health education and family planning programs would target urban dwellers, women who start sexual intercourse between the ages of 15 and 19, women with more than one sexual partner, women with a desire to limit childbearing, and women with poor contraceptive knowledge in order to reduce induced abortion.

**Supplementary Information:**

The online version contains supplementary material available at 10.1186/s12905-022-01695-0.

## Background

Abortion is the termination of a pregnancy before the 28th week of gestation after the last normal menstrual period or birth weight less than 1000gm [1]. Induced abortion is described as intentional medical or surgical termination of a live fetus before it is viable, and spontaneous abortions generally referred to as miscarriages, occur when an embryo or fetus is lost due to natural causes [2]. Globally, from 2015 to 2019, approximately 73.3 million induced abortions were performed annually, with 45% of these abortions being performed unsafely. Almost half of these unsafe abortions occurred in developing countries, including Ethiopia. Developing countries accounted for more than 98% of all unsafe abortions [3, 4]. About 210 million women become pregnant per year, with one out of every ten pregnancies ending in an unsafely induced abortion worldwide [5, 6]. Every year, an estimated 68,000 women die as a result of unsafe abortions around the world, with another 5.3 million suffering temporary or permanent disability. In developing countries, unsafe abortion is a leading cause of maternal mortality and morbidity. In addition to this, the public health burden is greatest in the developing world [7].

In Africa, unsafe abortion complications account for almost half of all maternal deaths [8]. Abortion-related maternal deaths account for 13% of all maternal deaths worldwide, most of which are caused by unsafe abortions [9]. In sub-Saharan Africa, more than 77% of induced abortions are terminated in unsafe conditions and account for 50% of maternal deaths, with the abortion rate in sub-Saharan Africa almost doubling from 4.3 million to 8.0 million between 1995–1999 and 2015–2019 [10, 11]. In East Africa, the annual abortion rate for all women of reproductive age is 34 per 1,000. Ethiopia has the world's fifth-highest rate of maternal mortality, with one in every twenty-seven women dying each year from pregnancy and childbirth complications [12]. Ethiopia is one of the low-income countries in sub-Sahara Africa with the highest maternal morbidity and mortality rates. The maternal mortality rate in Ethiopia was 412 maternal deaths per 100,000 live births, according to the 2016 Ethiopia Demographic and Health Survey (2016 EDHS) [13].

Around 3.27 million women became pregnant each year in Ethiopia, of which approximately half-million ends by abortion and the rate of abortion is highest in the urban area of Ethiopia, which is 92 per 1000 in Addis Ababa and 78 per 1000 in smaller regions like Harari and Dire Dewa [12, 14]. Previous studies in different parts of Ethiopia found that the prevalence of induced abortion was 42.7%, 18.8%, 12.3%, and 4% in the Harari region, Southwest Ethiopia, Guraghe Zone, and Gondar Town, respectively [6, 12, 14, 15]. In Ethiopia, abortion was responsible for 8.6% of maternal deaths [16]. Ethiopia made some strides in 2005 by revising the abortion law, which previously only allowed procedures to save a woman's life, and making safe abortion available to many women. Following that, abortion is legal whether the pregnancy is the result of rape or incest, the continuation of the pregnancy risks the mother's or child's health, the fetal disability is severe or incurable, and the woman is in a minority that is physically and mentally unprepared for childbirth [17].

Despite the presence of technological advancement in health care, unsafe abortion remains essentially unchanged globally as well as Ethiopian women are suffering from an increased risk of abortion-related complications, due to various reasons for example unmet family planning method need, rape, early sexual practice, etc. [18, 19]. Women seek induced abortion for a variety of reasons, depending on their circumstances. According to a study conducted in Denmark and Uganda, the strongest determinant of women's decision to have an abortion is being single, followed by being under the age of 19, having two or more children, being a student, or being unemployed [20, 21]. In Ethiopia, similar findings were revealed that unwanted pregnancy, single marital status, young age, low income, occupational status, the mother's level of education, is the major contributing factors to induced abortion [6, 14, 22].

Several efforts have been made to improve abortion-related services, including the construction of more health centers and the training of more mid-level healthcare providers, the expansion of abortion services to primary healthcare units, and the development and dissemination of national guidelines for providing legal and safe abortion services [23]. In 2014, the guideline was revised to update the gestational age limits for medication abortion and to make second-trimester abortion services more available [24]. As a result of these efforts, unintended pregnancy decreased to 38% in 2014 from 42% in 2008, abortion in health facilities increased to 53% in 2014 from 27% in 2008, and induced abortion and post-abortion care provided by mid-level providers increased to 53% in 2014 from 27% in 2008 [25].

Even with numerous initiatives and attempts to increase access to safe abortion facilities, nearly six out of ten abortions in Ethiopia are still conducted in a risky manner [26]. Despite the problem's significance, little is known about the factors that lead to women terminating their pregnancies. A further enhancement is still needed; so assessing its risk factors is important for tracking progress toward sustainable development goals. As a result, this study aims to identify determinants of induced abortion among women who received maternal health care services in public hospitals of Arba Minch and Wolayita Sodo town, southern Ethiopia, 2021.

## Methods

### Study setting, and period

This study was conducted at selected public hospitals of Arba Minch and Sodo town, Southern Ethiopia from April 15th–June 15th, 2021. Arba Minch and Wolayita Sodo are town found in the South Nations, Nationalities and Peoples’ Region (SNNPR) of Ethiopia. In the towns of Arba Minch and Wolayita Sodo, there are four hospitals, but the study was conducted in selected two public hospitals (Arba Minch General Hospital and Wolayita Sodo University Teaching & Referral Hospital). The total population of the Arba Minch town 112,724 among them 50.2% are females whereas Wolayita Sodo 250,521 among them 79,871 (52%) males, and 73,650 (48%) females. There are four public health facilities (one general hospital, one primary hospital and two health centers), thirty-two private medium and higher clinic, one Marie Stopes clinic, twelve drug stores, and two community pharmacies in Arba Minch town [27]. According to the medical director's report for the fiscal year 2020, Arba Minch General Hospital provides 100 comprehensive abortion care services, 195 antenatal care services, and 180 postnatal care services per month on average. There are two (one government and one private) hospital, three health centers, one Marie Stopes clinic and thirty private medium and higher clinics in Wolayita Sodo town [28]. According to the medical director's report for the fiscal year 2020, Wolayita Sodo teaching and referral Hospital provides 150 comprehensive abortion care services, 250 antenatal care services, and 210 postnatal care services per month on average. per month on average.

### Study design

An institutional based unmatched case–control study was conducted.

### Population

#### Source population

All women who received maternal health care services in the public hospitals of Arba Minch and Wolayita Sodo town.

#### Study population

All women who received comprehensive abortion care and those who visited maternal and child health (MCH) units for antenatal or postnatal care services in selected public hospitals of Arba Minch and Wolayita Sodo town during the data collection period.

### Selection of cases and controls

**Cases:** women who received induced abortion care services in selected public hospitals during the data collection period or who received post-abortion care services after being presented to the selected public hospital with an attempt of induced abortion.

**Controls:** women who had at least one pregnancy history in the last 12 months, and who came for maternal health care (antenatal or postnatal care) services in selected public hospitals and never had history of induced abortion.

### Exclusion criteria

Women presented to the selected public hospitals for spontaneous abortion care after diagnosed by physician and unable to communicate or seriously ill until the end of the data collection period were excluded.

### Sample size determination

For a case–control study, the sample size for this study was determined by using the Stat Cal application of Epi- Info version 7 software and two population proportion formulas. The following assumptions were considered: 95% level of confidence, 80% of power, the ratio of case to control 1:3 and percent of case exposed 8.2% and percent of control with exposure 21%. The percentages of cases and controls of exposure variable were taken from study conducted in Addis Ababa that the most determinate variables for induced abortion were monthly income [29]. Based on the above assumptions, the minimum estimated sample size for this study was 94 cases and 281 controls. After considering 10% of non-response rate, the final sample size for this study was 103 cases and 310 controls.

### Sampling technique and procedures

**Fig. 1:**
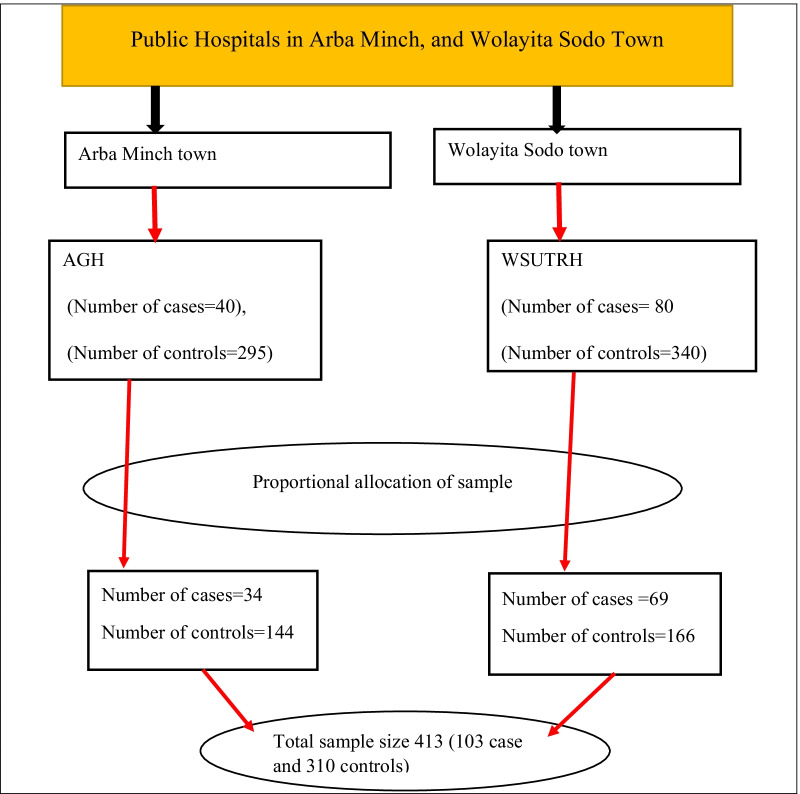
Schematic presentation of sampling procedures to identify the determinants of induced abortion among women received maternal health care services in public hospitals of Arba Minch and Wolayita Sodo town, Southern Ethiopia, 2021. Whereas *AGH* Arbaminch General Hospital, WSUTRH Wolayita Sodo University Teaching and Referral Hospital

In this study, Arba Minch general hospital and Wolayita Sodo University teaching & referral Hospital were included. By reviewing the previous year's two-month report in a data collection period, in Arba Minch General hospital, and Wolayita Sodo University teaching & referral hospital on average of 40, and 80 women received induced abortion care and 295 & 340 women received maternal health care services respectively. The proportional allocation method was used to include 413 women in the sample. The cases were selected using a consecutive sampling technique until the required sample size was reached. Controls were selected using a systematic random sampling technique. The lottery method was used to select the first control and then every second controls was interview until the required sample size was reached (Fig. 1).

### Study variables

Induced abortion was the dependent variable for this study. The independent variables were socio-demographic and economic factors (age, residence, marital status, occupational status, educational status, monthly income), reproductive and maternal health related factors (age at first marriage, age at first sexual encounter, multiple sexual partner, number of pregnancy, number of living children, pregnancy status), and contraceptive related factors (use, and knowledge).

### Operational definition and measurements

*Induced abortion*: intentional termination of pregnancy, by any means or person other than spontaneous [30]. *Contraceptive use*: the percentage of women who used any modern contraceptive methods prior to current pregnancy [31]. *Contraceptive knowledge*: based on bloom cut of point women who answered 75–100%, 50–74%, and < 50% correctly on six knowledge-assessment questions were considered to have good knowledge, moderate knowledge, and poor knowledge, respectively [32].

### Data collection tools and procedures

Data were collected using a standardized and pretested interviewer-administered questionnaire, which was adapted from previous related studies [22, 29, 31, 33]. The questionnaires contain three sections: Sociodemographic characteristics, reproductive and maternal health related, and contraceptive related factors (Additional file 1). Six BSc holder midwives were recruited to collect the data. In addition to this two supervisors who have MSc degree holders were recruited for supervisory activities. The principal investigator gave three days of theoretical and practical training to data collectors and supervisors on data collection tools, interview techniques, information confidentiality, and the objective and relevance of the study. The Kobo collect version 3.1 application was installed on the data collector's Android mobile, and the blank form was downloaded from the Kobo toolbox server. Two weeks before the actual data collection, the tool was pre-tested on 20 women (5 cases and 15 controls) in Chencha and Bodti primary hospitals. After obtaining respondent informed consent, an exit face-to-face interview and record review were used to collect data. Supervisors communicated on a regular basis with data collectors to ensure that the data collection procedure was followed. To avoid misclassification questionnaires gave a code for cases and controls (1 = cases, 0 = controls) and the women seeking maternal health care services were asked about a history of previous induced abortions and their medical records were reviewed to ensure they are true controls. Finally, on a weekly basis, the data collectors sent the filled questionnaire forms to the Kobo toolbox server.

### Data quality management

The questionnaire initially prepared in English and translated to the Amharic language, and then translated back to English by the expert to check the consistency. Data collectors and supervisors were trained for two days to become familiar with all types of data, tools, and data collection methods and objectives, as well as one day of practical sessions on Kobo Collect. A pre-test was conducted on 5% of the participants in Chencha and Bodti primary hospital, and any ambiguity, as well as the missed points, were added into the final version of the questionnaire. The supervisors checked completed questionnaires for key contents before uploading them from the Android mobile phone to the Kobo toolbox server to ensure data quality. All data were collected on-site using Android mobile devices and uploaded to the Kobo server on a weekly basis using Kobo collect version 3.1. The principal investigator also checked the sent files from each data collector on a regular basis for consistency and completeness.

### Data processing and analysis

The Kobo server data were downloaded as an excel file and exported to SPSS V.25 for cleaning, coding, ensuring completeness and accuracy, and then to Stata V.14 software for further analysis. A descriptive analysis was done to describe the pertinent characteristics of the study participants. After that, simple frequencies, percentages, and summary measures were computed. Both bivariate and multivariable analyses were used to assess the association between each independent variable and the outcome variable by using binary logistic regression. The goodness of fit was checked by Hosmer and Lemeshow test. Variables with a 95% confidence interval and P-value < 0.25 during the bivariate analysis were included in the multivariable logistic regression analysis in order to control all potential confounding variables. In addition, even if the above parameters were not being met, variables that were significant in previous studies and from a contextual viewpoint were included in the final model. Multi co-linearity was checked by co-linearity diagnostic statistics via variance inflation factors and tolerance test. Adjusted odds ratios with a 95% confidence interval were calculated and P-value less than 0.05 was considered as statistically significant. Finally, data were being presented using tables, graphs, and texts.

## Results

### Sociodemographic and economic characteristics

Out of 413 participants, 412 women completed the face-to-face interview with a response rate of 100% for cases and 99.7% for controls. The report encompasses 412 women of which 103 (25%) were cases while 309 (74.8%) were controls and it was unmatched case–control study.

The mean and standard deviation of respondents age were 25.7 ± 5 years (cases 24 ± 6.2 and controls 26.2 ± 4.5). The minimum age of cases was 15 years old and maximum age was 38 years old whereas the minimum and maximum age of controls was 16 and 40 years old respectively. Among the study participants, 28(27.18%) of the cases and 140(45.31%) of controls age ranges from 25 to 29 years. Of the respondents, 52(50.49%) cases and 155(50.16%) controls were Wolayita ethnicity. Seventy-six (73.79%) of the cases and 175 (56.63%) of controls were urban residents. Regarding marital status, around 41 (39.8%) of the cases and 306(99%) controls were married and 45(43.69%) of the cases and 151(48.87%) of controls were protestant religion followers.

Regarding educational status of women, 43(41.75%) of the cases and 90(29.13%) controls had secondary educational level. The overall median and interquartile range of monthly income was 62.5 (25 to 100) USD and 37.5 (interquartile range 12.5 to 90 USD) for cases whereas 62.5(30 to 107.5 USD) for controls. Out of the respondents, 62(60.19%) for cases and 130(42%) for controls were earned less than 50 USD per month. Related to the occupational status of respondents, 13(12.62%) of the cases and 106(34.3%) controls were housewife (Table1).

**Table 1:** Sociodemographic and economic characteristics of women who received maternal health care services in public hospitals of Arba Minch and Wolayita Sodo town, southern Ethiopia, 2021

Variables	Cases (*n* = *103*)	Controls (*n* = *309*)
**Respondent’s age (in years)**		
15–19	31(30.1)	20 (6.47)
20–24	22(21.36)	74 (23.95)
25–29	28(27.18)	140(45.31)
≥ 30	22(21.36)	75(24.27)
**Ethnicity**		
Wolayita	52(50.49)	155 (50.16)
Gamo	28(27.18)	111 (35.92)
Amhara	13(12.62)	16 (5.18)
Gofa	6(5.83)	13 (4.21)
Other*	4(3.88)	14 (4.53)
**Residence**		
Urban	76 (73.79)	175 (56.63)
Rural	27 (26.21)	134 (43.37)
**Marital status**		
Single	49 (47.57)	1 (0.32)
Married	41 (39.81)	306 (99.03)
Other**©**	13 (12.62)	2 (0.65)
**Religion**		
Orthodox	45 (43.69)	131 (42.39)
Protestant	45 (43.69)	151 (48.87)
Catholic	8 (7.77)	13 (4.21)
Muslim	5 (4.85)	14 (4.53)
**Educational status**		
No formal education	11 (10.68)	61 (19.74)
Primary	26 (25.24)	67 (21.68)
Secondary	43 (41.75)	90 (29.13)
Diploma and above	23 (22.33)	91 (29.45)
**Average monthly income(USD)**		
< 50 (low)	62 (60.19)	130 (42.07)
50–100 (medium)	22 (21.36)	98 (31.72)
≥ 101 (high)	19 (18.45)	81 (26.21)
**Occupational status**		
Government employee	23 (22.33)	68 (22.01)
Merchant	19 (18.45)	68 (22.01)
Student	34 (33.01)	27 (8.74)
Housewife	13 (12.62)	106 (34.30)
Other** ± **	14 (13.59)	40 (12.94)

*Tigre and Oromo, © separated, widowed and divorced, ± daily labor and unspecified

### Reproductive and maternal health characteristics

Fourteen (25.9%) of the cases and 64 (20.78%) of the controls were married before the age of 18. The overall mean and standard deviation of women age at first marriage was 21 ± 3.1 (cases 21.29 ± 3.6 and controls 21 ± 3). The minimum age at first marriage among cases was 16 years old and maximum age was 34 years old whereas the minimum and maximum age of first marriage among controls was 15 and 33 years old respectively.

Sixty-five (63.1%) of the cases and 118 (38.19%) of the controls age ranges from 15 to 19 at the time sexual debut. The mean and standard deviation of women age at first sexual encounter was 20 ± 2.99 years (cases 18.87 ± 3.2 and controls 20 ± 2.8). In both cases and controls, the minimum and maximum age at first sex were 15 and 28 years old, respectively. Forty-two (40.78%) of cases and 48 (15.53%) of controls had more than one sexual partners in past 12 months. Out of respondents, 42 (40.78%) of cases and 76 (24.6%) of controls had at least one pregnancy during their lifetime. Fifty-one (49.51%) of the cases and 61(19.74%) of controls had no history of delivery. Regarding number of alive children, 15(14.56%) of the cases and 130(42%) of the controls had one alive child.

**Fig. 2:**
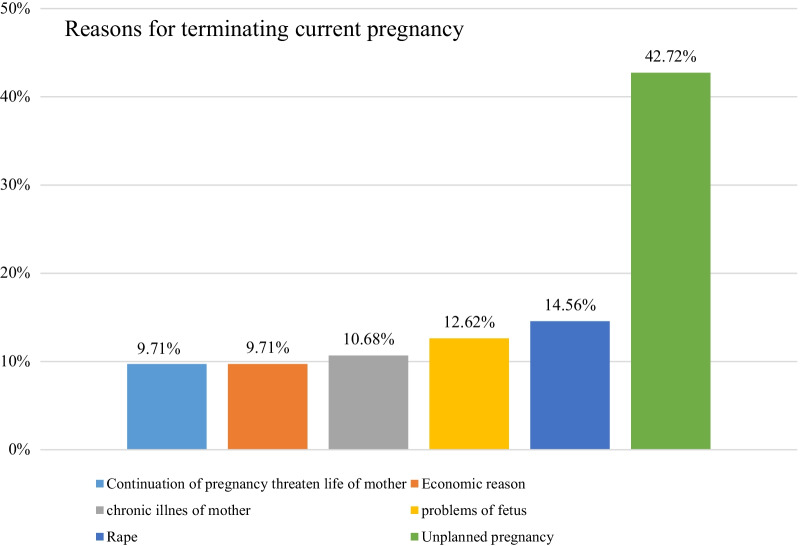
The reason for termination of current pregnancy among cases who received maternal health care services in public hospitals of Arba Minch and Wolayita Sodo town, southern Ethiopia, 2021

About 72 (69.9%) of cases and 33 (10.68%) of controls reported their last pregnancy was unplanned and Partner pressure (33.33%) and contraceptive failure (27.78%) were the most common reasons for unplanned pregnancy among cases. Whereas contraceptive failure was reported by more than half (54.55%) of the controls as the main reason for unplanned pregnancy. In terms of abortion complications, 43 (41.75%) of the cases and 164 (53.07%) of the controls had awareness about abortion-related complications. Of those who knew abortion complications, 31(30%) of cases and 106(34.3%) of controls were aware of at least two or more complications of induced abortion. Out of respondents, 35(33.98%) for cases and 100(32.36%) for controls had information about Ethiopian abortion law (Table 2). In the current studies, 44(42.72%) cases stated that their reason for termination was an unplanned pregnancy. Approximately 9.71% of cases terminated their pregnancy because the mother's life would be threatened if the pregnancy continued (Fig. 2).

**Table 2:** Reproductive and maternal health characteristics of women who received maternal health care services in public hospitals of Arba Minch and Wolayita Sodo town, southern Ethiopia, 2021

Variables	Cases (*n* = *103*)	Controls (*n* = *309*)
**Age at first sex**		
15–19	65 (63.11)	118 (38.19)
20–24	32 (31.07)	166 (53.72)
≥ 25	6 (5.83)	25 (8.09)
**Gravida**		
1	42 (40.78)	76 (24.60)
2	23 (22.33)	117 (37.86)
≥ 3	38 (36.89)	116 (37.54)
**Party**		
0	51 (49.51)	61 (19.74)
1	15 (14.56)	127 (41.10)
2	22 (21.36)	71 (22.98)
≥ 3	15 (14.56)	50 (16.18)
**Number of alive children**		
0	52 (50.49)	64 (20.71)
1	15 (14.56)	130 (42.07)
2	21 (20.39)	65 (21.04)
≥ 3	15 (14.56)	50 (16.18)
**Reason for unplanned pregnancy**		
Contraceptive failure	20 (27.78)	18 (54.55)
Forget to take contraceptive	12 (16.67)	4 (12.12)
Partner pressure	24 (33.33)	5 (15.15)
Don’t know any contraceptive	16 (22.22)	6 (18.18)
**Awareness of complications**		
Aware of at least two and more complications	31 (30.1)	106 (34.3)
Aware only one complication	12 (11.65)	58 (18.77)
Unaware of complication	60 (58.25)	145 (46.93)

### Contraceptive related characteristics

Eighty-seven (84.47%) of cases and 301 controls (97.41%) had heard about family planning methods. Regarding the source of information, 47(54%) of the cases and 183(60.8%) controls (60.8%), 29(33.33%) of cases and 58(19.27%) controls, & 11(12.64%) of cases and 60(19.93%) controls were heard from health facilities, mass media/printed materials, and family/friends respectively. Eighty-six (83.5%) of the cases and 297 (96.12%) of the controls were aware of at least one type of modern contraceptive. As a result, oral contraceptive pills 60 (69.77%) and Nexplanon 252 (84.85%) were the most commonly mentioned contraceptive methods by cases and controls, respectively. Eighty-two (79.61%) of the cases and 303 (98.06%) of the controls were aware of contraceptive methods used to prevent unwanted pregnancy, with the majority of 84 cases (84.55%) and 305 controls (98.7%) were aware of contraceptive methods used to space & a limited number of children. Eighty-one (78.64%) of cases & 291(94.17%) of controls had good knowledge about contraceptive methods (Table 3).

**Table 3:** Contraceptive related characteristics of women who received maternal health care services in public hospitals of Arba Minch and Wolayita Sodo town, southern Ethiopia, 2021

Variables	Cases (*n* = *103*)	Controls (*n* = *309*)
**Know any contraceptive methods**		
Yes	86 (83.50)	297 (96.12)
No	17 (16.50)	12 (3.88)
**Type of contraceptive methods do you know (*n* = *383*)**		
Emergency pills	23 (26.74)	85 (28.62)
Oral contraceptive pills	60 (69.77)	197 (66.33)
Condom	33 (38.37)	53 (17.85)
Nexplanon	56 (65.12)	252 (84.85)
IUCD	42 (48.84)	147 (49.49)
Other*	4 (4.65)	55 (18.52)
**History of contraceptive use**		
Yes	66 (64.08)	253 (81.88)
No	37 (35.92)	56 (18.12)
**Contraceptive method last used (*n* = *319*)**		
Emergency pills	9 (13.64)	8 (3.16)
Oral contraceptive pills	22 (33.33)	27 (10.67)
Injectable	15 (22.73)	109 (43.08)
Nexplanon	15 (22.73)	92 (36.36)
IUCD	2 (3.03)	9 (3.56)
Other*	3 (4.55)	8 (3.16)
**Reason for using contraceptive (*n* = *319*)**		
Preventing unintended pregnancy	49 (74.24)	95 (37.35)
Preventing STI	3 (4.55)	3 (1.19)
Helps to space & limit children	14 (21.21)	155 (61.26)
**Reason for not using contraceptive (*n* = *93*)**		
Fears of side effect	10 (27.03)	5 (8.93)
Against my religion	6 (16.22)	12 (21.43)
Opposition from my husband	2 (5.41)	5 (8.93)
Other®	19 (51.35)	34 (60.71)
**Knowledge about contraceptive**		
Good	81(78.64)	291 (94.17)
Moderate	9 (8.74)	14 (4.53)
Poor	13 (12.62)	4 (1.29

*Natural family planning methods (breast feeding, calendar methods), ®wants to have more children, and reason not specified

### Determinants of induced abortion

In binary logistics regression analysis, age, residence, educational status, monthly income, age at first intercourse, multiple sexual partners, number of alive children, and knowledge on contraceptives were found to be candidate variables for final multivariable logistics regression analysis model.

In the multivariable analysis, residence, age at first sex, multiple sexual partners, number of children, and knowledge of contraceptives were statistically associated with induced abortion. The odds of having an induced abortion were 2.33 times higher in women who lived in an urban area than women living in rural areas (AOR = 2.33, 95%CI: 1.26, 4.32). A woman who had encountered first sex at age of 20–24 years was 49% less likely to have an induced abortion as compared to women who encounter first sex at age of 15–19 years (AOR = 0.51, 95% CI:0.27,0.97). The odds of induced abortion were 5.47 times higher among women who had more than one sexual partner than those who had a single sexual partner (AOR = 5.47, 95%CI: 2.98, 10.03). Women who had one child were 68% less likely to have an induced abortion than those who had three or more children (AOR = 0.32, 95%CI: 0.10, 0.99). The odds of induced abortion were 88% less likely among women who had good knowledge of contraceptives than those who had poor knowledge of contraceptives (AOR = 0.12, 95%CI: 0.03, 0.46) (Table 4).

**Table 4:** Bivariate and multivariable analysis of determinants of induced abortion among women who received maternal health care services in public hospitals of Arba Minch and Wolayita Sodo town, southern Ethiopia, 2021

Variables	Cases	Controls	COR (95%CI)	AOR (95%CI)	P-value
**Respondent age groups**					
15–19	31(30.1%)	20(6.47%)	1	1	
20–24	22(21.36%)	74(23.95%)	0.19(0.09,0.40)	0.47(0.17,1.25)	0.13
25–29	28(27.18%)	140(45.31%)	0.12(0.06,0.25)	0.40(0.12,1.29)	0.13
≥ 30	22(21.36%)	75(24.27%)	0.18(0.90,0.39)	0.58(0.13,2.48)	0.47
**Residence**					
Urban	76(73.79%)	175(43.37%)	2.15(1.3,3.5)	2.33(1.26,4.32)*	**0.007**
Rural	27(26.21%)	134(56.63%)	1	1	
**Educational status**					
No formal education	11(10.68%)	61(19.74%)	1	1	
Primary	26(25.24%)	67(21.68%)	2.15(0.98,4.7)	1.44(0.53,3.93)	0.47
Secondary	43(41.75%)	90(29.13%)	2.6(1.26,5.54)	2.04(0.79,5.29)	0.14
Diploma and above	23(22.33%)	91(29.45%)	1.4(0.63,3.08)	1.32(0.48,3.66)	0.59
**Average monthly income(USD)**					
< 50	62(60.19%)	130(42.07%)	2.03(1.13,3.64)	1.10(0.47,2.55)	0.82
50–100	22(21.36%)	98(31.72%)	0.96(0.48,1.89)	0.78(0.34,1.74)	0.55
≥ 101	19(18.45%)	81(26.21%)	1	1	
**Age at first sex**					
15–19	65(63.11%)	118(38.19%)	1	1	
20–24	32(31.07%)	166(53.72%)	0.35(0.21,0.56)	0.51(0.27,0.97)*	**0.04**
25 ≥	6(5.83%)	25(8.09%)	0.43(0.17,1.11)	0.97(0.28,3.35)	0.95
**More than one sexual partner**					
Yes	42(40.78%)	48(15.53%)	3.7(2.27,6.16)	5.47(2.98,10.03)*	** < 0.001**
No	61(59.22%)	261(84.47%)	1	1	
**Number of alive children**					
Zero	52(50.49%)	64(20.71%)	2.7(1.36,5.36)	1.20(0.36,3.96)	0.95
One	15(14.56%)	130(42.07%)	0.38(0.17,0.84)	0.32(0.10,0.99)*	**0.04**
Two	21(20.39%)	65(21.04%)	1.07(0.50,2.29)	1.18(0.46,2.99)	0.72
Three & above	15(14.56%)	50(16.18%)	1	1	
**Knowledge on contraceptive**					
Poor	13(12.62%)	4(1.29%)	1	1	
Moderate	9(8.74%)	14(4.53%)	0.19(0.04,0.80)	0.35(0.06,1.85)	0.22
Good	81(78.64%)	291(94.17%)	0.08(0.02,0.26)	0.12(0.03,0.46)*	**0.002**

The bold font indicates statistical significant

*Significant at P-value < 0.05

## Discussion

Inducing abortion is one of the major public health concerns in the world today. Maternal mortality and morbidity continue to be alarmingly high. Sub-Saharan Africa has the highest estimated proportion of induced abortions, and women are more likely to die as a result of abortion than any other region of the world [10]. Identifying the determinants of induced abortion is crucial for many sub-Saharan African countries like Ethiopia. Thus, this study revealed a facility-based case–control study to identify factors associated with induced abortion in the study settings. Among characteristics assessed in this study; residence, age at first sex, multiple sexual partners, number of children, and contraceptive knowledge had a significant association with induced abortion.

This study showed that urban residents were more likely to report having an induced abortion compared with those who were rural residents. This finding was supported by studies done in different parts of Ethiopia [15, 34–36]. This finding was also consistent with a study conducted in Ghana [37]. This may be due to the fact that women who live in urban are exposed to a variety of factors (like peer pressure, drinking too much alcohol, unprotected sex) that make them vulnerable to risky sexual behaviors that result in an unwanted pregnancy. Another possible explanation is that women in urban areas have more access to abortion services than women in rural areas. Similarly, those living in urban areas are more likely to have premarital sex, which can lead to unintended pregnancy and induced abortion. The finding of this study is supported by the findings of the 2016 EDHS, which revealed that urban residents have a higher rate of premarital sex than their rural counterparts [38].

As indicated in this study, women who had their first sex start between the ages of 15 and 19 were more likely to have induced abortion than those who had their first sexual intercourse at the age of 20 or later. Similarly, other studies from Ethiopia [29], and Chile [39] have found that women who had their sexual debut at a younger age are more likely to have an induced abortion. This could be due to adolescents having unstable marital relationships, not completing their education, and lack of knowledge about safe sex practices, including how to prevent unintended pregnancy and other reproductive health problems. Another possibility is that women who had their first sexual encounter at a younger age may have limited knowledge on how to use family planning. Multiple sexual partners were found to be significant determinants of induced abortion in this study. Women who had more than one sexual partner were found to have increased odds of having an induced abortion than women who had a single sexual partner. This finding was consistent with studies done in Ethiopia [35, 36], and Cambodia [40]. This could be due to the fact that having multiple sexual partners leads to women being in unstable marital relationships, which leads to irregular contraceptive use that also leads to contraceptive failure and unwanted pregnancy.

In this study, women who had one child were less likely to report having an induced abortion as compared with women who had three or more children. This finding was supported by studies done in Ethiopia [6, 29], Nepal [41], and Iran [42, 43]. This is obvious that women who had several pregnancies would have the tendency not to have additional children so they may induce their current pregnancy to avoid unwanted pregnancies.

Women who had good knowledge of contraceptives were less likely to have an induced abortion than those who had poor knowledge of contraceptives. This finding was congruent with studies done in Ethiopia [44], Ghana [31], and Tanzania [45]. This could be due to the fact that women who have inadequate knowledge of contraceptives are unable to prevent themselves from unintended pregnancies and thus seek induced abortion to avoid having unwanted children.

The strength of this study was used primary data by directly interviewing study participants, and incident cases were used to reduce the problem of establishing a temporal relationship. Since this study was conducted at a general and teaching hospital with an experienced obstetrician and gynecologist, there was no misclassification between spontaneous and induced abortion among cases. The cases were included in this study after being diagnosed by a physician.

### Limitation of the study

However, the study has some limitations. There is a concern of information bias because women's history of induced abortion was self-reported, so some controls may have had abortions in the past but wanted to give responses. The medical records of controls were cross-checked to ensure they had not obtained an abortion. Secondly, this study was also prone to recall bias, as respondents may not have been able to recall past events accurately. Thirdly, non-governmental and private health care facilities were not included in this study. Finally, because the study was conducted in a health facility, the findings may not be generalizable to the general population of women.

## Conclusions

Women with urban residents, early sexual initiation, having multiple sexual partners, poor knowledge of contraceptives and those with a higher number of children in the household were independently associated factors with induced abortion. As a result, interventions focusing on those identified factors by the concerned bodies could probably reduce the burden and consequences of induced abortion. Sexual and reproductive health education and family planning programs should target community-based outreach programs regularly to raise community awareness of contraception by involving adolescents and the prevention of unintended pregnancy to reduce induced abortion.

## Supplementary Information

**Additional file 1.** English version information sheet. Amharic version information sheet and consent form.

## Data Availability

The datasets generated and /or analyzed during the current study are not publicly available due to preserving participant anonymity but are available from the corresponding author on reasonable request (Mesfin Abebe, mesfiaau@gmail.com).

## Abbreviations

COVID-19: Coronavirus Disease 2019
EDHS: Ethiopian Demographic and Health Survey
IUCD: Intra-uterine contraceptive device
MCH: Maternal and child health
AGH: Arbaminch General Hospital
WSUTRH: Wolayita Sodo University Teaching and Referral Hospital
