# Supplementary material for: Determinants of induced abortion among women received maternal health care services in public hospitals of Arba Minch and Wolayita Sodo town, southern Ethiopia: unmatched case–control study
Source: BMC Womens Health. 2022 Apr 9;22:107. doi: 10.1186/s12905-022-01695-0 (PMC8994190; doi:10.1186/s12905-022-01695-0)
Supplement: Supplementary file 1 — Additional file 1. English version information sheet. Amharic version information sheet and consent form. [file 12905_2022_1695_MOESM1_ESM.docx]

**English version information sheet**

Good morning/afternoon dear participant! My name is-------------, I am working as a data collector for the study being conducted in this hospital on determinants of induced abortion among women receiving maternal health care services in public hospitals of Arba Minch and Wolayita Sodo town, Southern Ethiopia by Mesfin Abebe, who is studying for his Master’s Degree at Arba Minch University, College of Medicine and Health Sciences, School of Nursing in Maternity and reproductive health. I respectfully request that you give me your time to clarify the research and why your institution was chosen as the study venue.

**The study title:** Determinants of induced abortion among women receiving maternal health care services in public hospitals of Arba Minch and Wolayita Sodo town, Southern Ethiopia, 2021.

**Purpose of study: -** The finding of this study provides information on determinant factors of induced abortion and helps health care providers, health extension workers, and local health departments to identify women who are more at risk of induced abortion. This may help them to outline specific priorities targets for eliciting local evidence-based strategies or intervention for preventing unwanted pregnancies and induced abortion. Moreover, the aim of this study is to write a thesis as a partial fulfilment of a master’s program in Master of Maternal and Reproductive Health Nursing for the principal investigator.

**Procedure: -** Arba Minch University will process the permission to each health institutions. Face-to-face interviews and record review will be used to collect data. As a participant, I respectfully request your permission to collect data for this study.

**Risk and benefits:** - Participating in this study poses no significant risk and requires just a few minutes of your time. Involvement in this research will not be paid in any way. However, the results of this study can provide valuable information for health institutions, health officials, and policymakers to fill in the gaps identified by this study.

**Confidentiality:** - The information you give me will be handled privately. There will be no information that can be used to identify you. The study's results will be general for the study area and will not provide any information about specific participants. The questionnaire will be coded to prevent names from being known. There will be no mention of the study in oral or written reports that could attach participants to it.

**Right to refusal/withdrawal: -** You have total autonomy on whether or not to participate in this study. You will have total freedom to withdraw from this study at any time.

**Contact address: -** The Arba Minch University ethical committee will review and approve this research project. If you have any questions, you can contact the person listed below and ask them whenever you want?

**Investigator**:-Mesfin Abebe

Phone contact /+251914141050/

E-mail/ [mesfiaau@gmail.com](mailto:mesfiaau@gmail.com)

Institutional Research Ethics Review Board (IRB), College of Medicine and Health Sciences, Arba Minch University: office phone number/+251461810221/

E-mail/irb.amu@yahoo.com/

**Declaration of informed voluntary consent**: I've read/will read the participant information sheet to me. I have clearly understood the purpose of the research, procedures, risks and benefits, confidentiality issues, participation rights, and contact address for any queries. I have been given the opportunity to ask questions about things that may have been unclear. I shall be informed that I have the right to withdraw from the study at any time or not to answer any question that I do not wish to have. Therefore, I declare my voluntary consent to participate with my initials in this study (signature).

Participant code -----------------------------------------------signature------------date-----------

Name of data collector--------------------------------------------signature------------date------------

**English version questionnaire form**

Name of data collector--------------------------------------------signature------------

Supervisory name--------------------------------------------------signature ----------

Questionnaire code------------------------------------------------signature-----------

Date of interview (day/month/year) -----------------------------

Name of health institution --------------------------------------

Status of respondents 1=cases, 0=controls

**Part I: Sociodemographic and Socioeconomic Characteristics**

| **S.No** | **Questions** | **Response and coding** | **Skip** |
| --- | --- | --- | --- |
| 101 | What is your Age? | _________ years |  |
| 102 | What is your Ethnicity? | 1. Gamo 5. Tigre 2. Gofa 6. Oromo 3. Woliyta 7.others(please specify) 4. Amhara --------------- |  |
| 103 | What is your Residence? | 1. Urban 2. Rural |  |
| 104 | Marital status of a participant | 1. Single 4. Widowed 2. Married 5. Separated 3. Divorced 6. Others please specify |  |
| 105 | What is your Religion? | 1. Orthodox 4. Catholic 2. Muslim 5. Others specify 3. Protestant ------------ |  |
| 106 | Educational status of a participant | 1. No formal education 2. Primary 3. Secondary 4. Diploma and above |  |
| 107 | Average income per monthly | ----------------------in birr |  |
| 108 | What is your current occupation? | 1. Government employee 4. Housewife 2. Merchant 5. Unemployed/jobless 3. Student 6. Daily labor   7. Others please specify…. |  |

**Part II: Reproductive and Maternal Health Characteristics**

| **S.No** | **Questions** | **Response and coding** | **Skip to** |
| --- | --- | --- | --- |
| 201 | How old were you during your first marriage? | ------- | If Q104 answer single, skip to 202 |
| 202 | How old were you during your first sexual encounter? | ------- |  |
| 203 | Do you have more than one sexual partner? | 1. Yes 2. No |  |
| 204 | Total number of pregnancy | -------in numbers |  |
| 205 | Total number of delivery | -------in numbers |  |
| 206 | Number of alive children | ------- in numbers |  |
| 207 | Was this/last pregnancy planned / wanted? | 1. Yes 2. No | If yes skip to Q208 |
| 208 | If no Q207, What is the cause of your unwanted pregnancy? | 1. Contraceptive failure 2. Forget to take contraceptive 3. Partner pressure 4. Don’t know any contraceptive 5. Others…….. |  |
| 209 | What is your reason for terminating this pregnancy? | 1. Unplanned pregnancy 2. Problem of the fetus 3. Chronic illness of the mother 4. Continuation of pregnancy threatens the life of the mother 5. Rape 6. Economic reason 7. Others…….. | If status of respondent=1 |
| 210 | Do you know any induced abortion-related complications? | 1. Yes 2. No | If no skip to Q 211 |
| 211 | If yes Q 210, What do you know about the complications of induced abortion? | 1. Bleeding 2. Infection 3. Death may be happen 4. Others…. |  |
| 212 | Do you have information about Ethiopian abortion law? | 1. Yes 2. No |  |

**Part III: Contraceptive Related Questions**

| **S.No** | **Questions** | **Response and coding** | **Skip to** |
| --- | --- | --- | --- |
| 301 | Have you ever heard about contraceptive methods? | 1. Yes 2. No | If no, skip to Q 302 |
| 302 | If yes Q 301, What was the source of information? | 1. Health facilities 2. Mass media/printed materials 3. Family/friend 4. Others please specify…. |  |
| 303 | Do you know about any contraceptive methods? | 1. Yes 2. No | If no, skip to Q 304 |
| 304 | If yes Q 303, what contraceptive methods do you know? (multiple responses are possible) | 1. Emergency pills 2. Regular pills 3. Condom 4. Implants 5. IUCD 6. Others, please specify…. |  |
| 305 | Do you know contraceptives used to prevent unwanted pregnancy? | 1. Yes 2. No |  |
| 306 | Do you know contraceptives used to spacing and limiting children? | 1. Yes 2. No |  |
| 307 | Have you ever used contraceptive before your current pregnancy? | 1. Yes 2. No | If no, skip to Q309 |
| 308 | If yes Q 307, What contraceptive methods have you last used? | 1. Emergency pills 2. Oral contraceptives pills 3. Injectable 4. Implants 5. IUCD 6. Others, please specify …. |  |
| 309 | What is your reason for using the contraceptive method? | 1. Preventing unintended pregnancy 2. Preventing sexual transmitted infection/disease 3. Helps to space children 4. Others, please specify |  |
| 310 | What are your reasons for not using contraceptives? | 1. Against my religion 2. Opposition from my husband 3. Fear of side effect 4. Wants to have more children 5. Others, please specify……. |  |

**Thanks so much for your participation!!!**

**Amharic version information sheet and consent form**

**ጤና ይስጥልኝ!!**

ስሜ ------------- እባላለሁ፡፡ እኔ በዚህ ሆስፒታል ውስጥ ጥናት እያካሄዱ ለሚገኙት የአቶ መስፍን አበበን በአርባ ምንጭ ዩኒቨርሲቲ የሁለተኛ ዲግሪ የትምህርት መሰናዶ ለማጠናቀቅ የመመረቂያ ጥናት በተመለከተ መረጃ ሰብሳቢ ነኝ ጥናቱን ለማብራራት እና ተቋምዎ ለምን የጥናት ቦታ ሆኖ እንደተመረጠ ጊዜዎን እንዲሰጡኝ በአክብሮት እጠይቃለሁ ፡፡

**የጥናቱ ርዕስ፡**-በአርባ ምንጭ እና ወላይታ ሶዶ ከተማ በሚገኙ በተመረጡ የህዝብ ሆስፒታሎች ላይ የእናቶች ጤና አጠባበቅ አገልግሎት በሚቀበሉ ሴቶች ላይ ፅንስ ማስወረድን/ማቋርጥን የሚወስኑ ምክንያቶችን በተመለከተ

**የጥናቱ ዋና አላማ፡** የዚህ ጥናት ዋና አላማ ሴቶች ፅንስ የሚያቋርጡበትን ዋና ምክንያቶች ምን እንደሆነ ለማወቅ ሲሆን ይህ በአካባቢያዊ ማስረጃ ላይ የተመሰረቱ ስልቶችን ወይም ያልተፈለገ እርግዝናን እና ፅንስ ማስወረድን ለመከላከል ይርዳል፡፡በተጨማሪም የዚህ ጥናት ዓላማ ለዋና ተመርማሪው በእናቶች እና በሥነ ተዋልዶ ጤና ማስተር ፕሮግራም ድህረ ምረቃ ማሟያ የሚሆን ነው፡፡

**የጥናቱ ጥቅም እና ጉዳት፡-** በዚህ ጥናት ውስጥ መሳተፍ ምንም አይነት ጥቅምም ሆነ ክፍያ አይኖርም ካለዎት ጊዜ ላይ ጥቂት ደቂቃዎችን ብቻ ይፈልጋል፡፡ ነገር ግን በእያንዳንዱ ጥያቄ ላይ የእርስዎ ታማኝነት እና ትክክለኛነት መልስ የጥናቱ ዓላማ ከግብ ለማድረስ ከፍተኛ ሚና ይኖረዋል፡፡

**የጥናቱ ሚስጥራዊነት፣** የምትሰጡት መረጃ በግል እና በጥንቃቂ ይካሄዳል ፡፡ እርስዎን ለመለየት የሚያገለግል ምንም መረጃ አይኖርም ፡፡ መጠይቁ ስሞች እንዳይታወቁ ለመከላከል ኮድ ይደረጋል ፡፡ ጥናቱን ተሳታፊዎችን ሊያገናኝ በሚችል የቃል ወይም የጽሑፍ ዘገባዎች ውስጥ ስለ ጥናቱ መጠቀስ አይቻልም **፡፡** የተሰበሰበዉም መረጃ ከጥናቱ ዋና ተመራማሪ እና ኣማካሪዎቹ በስተቅር ለሌላ ለማንኛውም ሰው ግልጽ አይሆንም፡፡ የተገኘዉም መረጃ ለታቀደለት ኣላማ ብቻ ይዉላል፡፡

**ያለመሳተፍ መብት፡** በዚህ ጥናት ውስጥ ለመሳተፍ ወይም ላለመሳተፍ አጠቃላይ በራስ ፍላጎትዎ ላይ የተመሰረተ ነው፡፡ ፡፡ በማንኛውም ጊዜ ከዚህ ጥናት መቀጠል ካልተመቸወት የማቋረጥ ነፃነት ይኖርዎታል ፡፡

**ልያገኙዋቸዉ የምችሉ ሰዎች**፡-የዚህ ምርምር ፕሮጀክት በአርባ ምንጭ ዩኒቨርሲቲ ተቋማዊ የምርምር የስነ-ምግባር ግምገማ ኮሚቴ ታይቶ የሚፀድቅ ይሆናል፡፡ የትኛዉንም ኣይነት ጥያቄ መጠየቅ ቢፈልጉ ከዚህ ቀጥለዉ የተጠቀሱትን ግለ-ሰቦች በማንኛዉም ጊዜ ማግኘትና መጠየቅ ይችላሉ፡፡

1. **መስፍን አበበ፡-**አርባ ምንጭ ዩንቨርሲቲ ፣ ተማሪ እና ዋና ተመራማሪ

**ስልክ ቁጥር/+251914141050/**

**ኢ-ሜይል/mesfiaau@gmail.com/**

1. ተቋማዊ የምርምር ሥነ-ምግባር ግምገማ ቦርድ ቢሮ፣የህክምና እና ጤና ሣይንስ ኮሌጅ፣አርባ ምንጭ ዩንቨርሲቲ

ቢሮ ስልክ ቁጥር /+251461810221/

ኢ-ሜይል/irb.amu@yahoo.com/

**በመረጃ የተደገፈ የስምምነት መግለጫ ቅጽ**

እኔ ከዚህ በላይ የተገለፀውን የተሳታፊውን መረጃ ወረቀት አንብቤአለሁ / አነባለሁ፡፡የምርምርውን ዓላማ ፣ አሰራሮችን ፣ አደጋዎችን እና ጥቅሞችን ፣ ሚስጥራዊነት ጉዳዮችን ፣ የተሳትፎ መብቶችን እና የማንኛውም አድራሻ በሚገባ ተረድቻለሁ፡፡ እኔ የምሰጠው መረጃ ለዚህ ጥናት አገልግሎት ብቻ የሚውል መሆኑንና ሚስጥር እንደሚጠበቅ እንዱሁም ማንነቴ እንደማይገለጽ ተነግሮኛል፡፡ ግልጽ ባልሆኑ ነገሮች ላይ ጥያቄዎችን ለመጠየቅ እድሉ ተሰጥቶኛል ::በተጨማሪም በጥናቱ መሳተፍ አለመሳተፍ ወይም ለማንኛውም ጊዜ ከጥናቱ ተሳታፊነት አቋርጬ መውጣት እንደሚችል ሙሉ መብት እንዳለኝ ተረድቻለሁ፡፡ በዚህ ጥናት በመሳተፌ ምንም ጉዳትም ሆነ የተለየ ጥቅም አንደማይሰጠኝ የተረዳሁ ስሆን ይህን ቃለ-መጠይቅ ለመጠየቅ ፍቃደኛ መሆኔን በፊርማዬ አረጋግጣለሁ፡፡ ለመቀጠል ፍቃደኛ ነዎት? አዎ ( ) አይደለሁም ( )

የተሳታፊ ፊርማ ________ ቀን (በኢትዮጵያ ኣቆጣጠር), __________________

የመረጃ ሰብሳቢ ሰም_________________________ ፊርማ_________________

የተቆጣጣሪ ስም--------------------------------------------------ፊርማ ----------

የጥያቄ ኮድ------------------------------------------------

የጤና ተቋም ስም --------------------------------------

የቃለ መጠይቁ ተሳታፊዎች ሁኔታ 1=ኪዝ, 0=ኮንትሮል

**Part I: ማህበራዊ-ስነ-ህዝብ እና ማህበራዊ-ኢኮኖሚያዊ ሁኔታ**

| **ተ.ቁ** | **ጥያቄዎች** | **ምላሽ እና ኮድ** | **ይዝለሉ** |
| --- | --- | --- | --- |
| 101 | እድሜዎ ስንት ነው? | _________ አመት |  |
| 102 | የእርስዎ ብሄር ምንድን ነው? | 1. ጋሞ 5. ትግሬ 2. ጎፋ 6. ኦሮሞ 3. ወላይታ 7. እባክዎን ይግለጹ……) 4. አማራ |  |
| 103 | መኖሪያዎ ምንድን ነው?? | 1. ከተማ 2. ገጠር |  |
| 104 | የጋብቻ ሁኔታ | 1. ያላገባች 2. ያገባች 3. የተፋታች 4. የትዳር አጋር የሞተባት 5. ከትዳር አጋር ጋር ተለያይተው የሚኖሩ 6. ሌላ ካለ ይግለፁ |  |
| 105 | ሃይማኖትዎ ምንድን ነው? | 1. ኦርቶዶክስ 4. ካቶሊክ 2. ሙስሊም 5. ሌላ ካለ ይግለጹ………. 3. ፕሮቴስታንት |  |
| 106 | የትምህርት ሁኔታ | 1. መደበኛ ትምህርት ያልተማረ 2. የመጀመሪያ ደረጃ ያጠናቀቀ 3. ሁለተኛ ደረጃ ያጠናቀቀ 4. ዲፕሎማ እና ከዚያ በላይ |  |
| 107 | ወርሃዊ ገቢ በአማካኝ | ---------------------ብር |  |
| 108 | የሥራ ሁኔታዎ ምንድን ነው? | 1. የመንግስት ሰራተኛ 2. ነጋዴ 3. ተማሪ 4. የቤት እመቤት 5. ሥራ አጥ 6. የቀን ሰራተኛ 7. ሌላ ይግለጹ -------- |  |

**Part II: የስነ-ተዋልዶ እና የእናቶች ጤና ሁኔታ**

| **ተ.ቁ** | **ጥያቄዎች** | **ምላሽ እና ኮድ** | **ይዝለሉ** |
| --- | --- | --- | --- |
| 201 | በመጀመሪያ ጋብቻዎ ጊዜ ዕድሜዎ ስንት ነው? | ------- | ለጥ.ቁ 104 መልስዎ ያላገባች ከሆነ ወደ ጥ.ቁ 202 ይዝለሉ |
| 202 | በመጀመሪያ የግብረ ሥጋ ግንኙነት ጊዜ ዕድሜዎ ስንት ነው? | ---------- |  |
| 203 | ከአንድ በላይ የወሲብ ጓደኛ አለዎት? | 1. አዎ 2. አይ |  |
| 202 | እስከ አሁን ለምን ያህል ጊዜ አርግዘዋል? (በቁጥር ይገለጽ) | ------በቁጥር |  |
| 203 | በህይወት የተወለዱት ልጆት ብዛት(በቁጥር  ይገለጽ) | ------በቁጥር |  |
| 204 | አሁን በህይወት ያሉት የልጆች ብዛት (በቁጥር ይግለፁ) | ------ በቁጥር |  |
| 206 | ይህ እርግዝና በእቅድ የተከሰተ/የተፈለገ ነበር? | 1. አዎ 2. አይ | መልሱዎ አዎ ከሆነ ወደ ጥ.ቁ 208ይዝለሉ |
| 207 | ያልተፈለ እርግዝና ያጋጠመዎት ምክንያትዎ ምንድነው? | 1. የእርግዝና መከላከያ ዘዴ አለመሳካት 2. የእርግዝና መከላከያ ዘዴን መውሰድ መርሳት 3. የጎደኛ ግፌት 4. ማንኛውንም የእርግዝና መከላከያ ዘዴን ስለማላውቅ 5. ሊላ ካለ ይጥቀሱ……… |  |
| 208 | ይህን እርግዝናን ለማቋረጥ ምክንያትዎ ምንድን ነው? | 1. ያልታቀደ እርግዝና 2. የፅንሱ ችግር 3. ስር የሰደደ የእናት ህመም 4. የእርግዝና መቀጠል የእናትን ሕይወት አደጋ ላይ ስለሚጥል 5. አስገድዶ መድፈር 6. ኢኮኖሚያዊ ምክንያት 7. ሌላ ካለ ይጥቀሱ……… |  |
| 209 | ከፅንስ ማስወረድ ጋር የተዛመዱ ውስብስብ ችግሮችን ያውቃሉ? | 1. አዎ 2. አይ | መልሱዎ አይ ከሆነ ወደ ጥ.ቁ 211ይዝለሉ |
| 210 | ከፅንስ ማስወረድ ውስብስብ ችግሮች የትኛውን ያውቃሉ? | 1. ደም መፍሰስ 2. ኢንፌክሽን 3. ሞት ሊሆን ይችላል 4. ሌላ ካለ ይጥቀሱ……… |  |
| 211 | ስለኢትዮጵያ ፅንስ ማስወገጃ ሕግ መረጃ አለዎት? | 1. አዎ 2. አይ |  |

**Part III: ከእርግዝና መከላከያ ጋር የተያያዙ ጥያቄዎች**

| **ተ.ቁ** | **ጥያቄዎች** | **ምላሽ እና ኮድ** | **ይዝለሉ** |
| --- | --- | --- | --- |
| 301 | ስለ የወሊድ መከላከያ ዘዴዎች ሰምተሽ ታውቃለሽ? | 1. አዎ 2. አይ | መልሱዎ አይ ከሆነ ወደ ጥ.ቁ 303 ይዝለሉ |
| 302 | የእርግዝና መከላከያ መረ ጃዉን ከ የ ት አ ገ ኙት? | 1. ጤና ተቋም  2. ሚድያ/ ጋዜጣ  3. ቤተሰብ/ ጓደኛ  4. ሌላ ካለ ይግለጹ……….. |  |
| 303 | ስለማንኛውም የእርግዝና መከላከያ ዘዴዎች ያውቃሉ? | 1. አዎ 2. አይ | መልሱዎ አይ ከሆነ ወደ ጥ.ቁ 305 ይዝለሉ |
| 304 | የትኛውን የእርግዝና መከላከያ ዘዴዎች ያውቃሉ? (ብዙ ምላሾች መመለስ ይቻላል) | 1. ድንገተኛ ክኒን 2. መደበኛ ክኒን 3. ኮንዶም 4. የ ክንድ ቆዲ ስር የሚቀበር ኢምፕላንት 5. በ ማህጸን ዉስጥ የሚቀመጥ ሎፕ 6. ሌላ ካለ ይግለጹ…. |  |
| 305 | የእርግዝና መከላከያ ያልተፈለገ እርግዝናን እንደሚከላከል ያውቃሉ? | 1. አዎ 2. አይ |  |
| 306 | የእርግዝና መከላከያ አራርቆ ለመውለድ ወይም የቤተሰብ ብዛትን ለ መገደብ እንደሚጠቅም ያውቃሉ | 1. አዎ 2. አይ |  |
| 307 | ከአሁኑ እርግዝና በፊት የእርግዝና መከላከያ ተጠቅመው ያውቃሉ? | 1. አዎ 2. አይ | መልሱዎ አይ ከሆነ ወደ ጥ.ቁ 309 ይዝለሉ |
| 308 | ለመጨረሻ ጊዜ ምን ዓይነት የወሊድ መከላከያ ዘዴዎችን ተጠቅመዋል? | 1. ድንገተኛ ክኒን 2. በአፍ የሚወሰድ ክኒን 3. በመርፌ የሚሰጥ 4. ቆዳ ስር የሚቀበር 5. ሎፕ 6. ሌሎች እባክዎን ይግለጹ… |  |
| 309 | የእርግዝና መከላከያ ዘዴን ለመጠቀም የእርስዎ ምክንያት ምንድነው? | 1.ያልታሰበ እርግዝናን መከላከል  2. በግብረ ሥጋ ግንኙነት የሚተላለፍ ኢንፌክሽን / በሽታን መከላከል  3.ልጆችን አራርቆ ለመውለድ ይረዳል  4.ሌሎች እባክዎን ይግለጹ |  |
| 309 | የእርግዝና መከላከያ ዘዴዎችን ላለመጠቀም የእርስዎ ምክንያት ምንድነው? | 1.በሃይማኖቴ ስለማይፈቀድ  2. ከባለቤቴ ተቃውሞ  3.የጎንዮሽ ጉዳትን በመፍራት  4. ብዙ ልጆች መውለድ ስለምፈልጋል  5. ሌሎች ካሉ እባክዎን ይግለጹ |  |

**ስለ ተሳትፎዎ በጣም እናመሰግናለን !!!**
